# Myopia: why the retina stops inhibiting eye growth

**DOI:** 10.1038/s41598-022-26323-7

**Published:** 2022-12-15

**Authors:** Barbara Swiatczak, Frank Schaeffel

**Affiliations:** 1 Institute of Molecular and Clinical Ophthalmology Basel (IOB), Basel, Switzerland; 2 Section of Neurobiology of the Eye, Ophthalmic Research Institute, University of Tuebingen, Tuebingen, Germany; 3 Zeiss Vision Lab, Ophthalmic Research Institute, University of Tuebingen, Tuebingen, Germany

**Keywords:** Neuroscience, Translational research

## Abstract

In myopia, the eye grows too long, and the image projected on the retina is poorly focused when subjects look at a distance. While the retina normally controls eye growth by visual processing, it seems to give up during myopia development. But what has changed? To determine whether the sharp image is in front or behind the retinal plane, a comparison of image sharpness in red and blue would provide a reliable cue because focal planes are about 1.3 D apart due to longitudinal chromatic aberration (LCA). However, up to now, it could not be demonstrated that the retina does, in fact, such a comparison. We used a new approach: movies were digitally filtered in real time to present either the blue channel of the RGB color format unfiltered while green and red were blurred (“blue in focus”), or the red channel was unfiltered while green and blue were blurred (“red in focus”) accordingly to the human LCA function. Here we show that, even though filtered movies looked similar, eyes became significantly shorter when the movie was sharp in the red plane but became longer when it was presented sharp in the blue plane. Strikingly, the eyes of young subjects who were already myopic did not respond at all—showing that their retina could no longer decode the sign of defocus based on LCA. Our findings resolve a long-standing question as to how the human retina detects the sign of defocus. It also suggests a new non-invasive strategy to inhibit early myopia development: keeping the red image plane on a computer screen sharp but low pass filtering the blue.

## Introduction

In the past decades, myopia became the most frequent developmental disorder in the eyes of young people in many countries of the world, now affecting more than two billion individuals^1^. In addition to poor visual acuity at a distance, the exaggerated expansion of the eyeball leads to progressive thinning of the tissue layers in the back of the eye which increases the risk of retinal degeneration, retinal detachment, and other ocular pathologies that may lead to blindness already in the mid of the life span^2^. Myopia typically starts to develop at school age but tends to progress during the following years. Why the eye deviates from its normally perfectly controlled growth path is intensively studied, but largely this question is still unresolved. Extended periods of near work and reading were traditionally associated with myopia development but a causal link or an explanation of a potential mechanism is lacking until today (reviews:^3,4^). A major step in the understanding of myopia was that axial eye growth is controlled almost exclusively by the retina^5,6^. Even more striking, it was found in the chicken that the retina is able to detect the sign of defocus also when only one viewing distance is available and the retinal image is heavily blurred^6–8^.

Less time spent outdoors is one of the main risk factors of myopia development. It has been shown in prospective study that children who spent outdoors 5 h more per week remained non-myopic when compared with children who became myopic^9^. The obvious difference between indoor and outdoor environments is light intensity. And indeed, it has been shown that exposure to bright light as found outdoors can delay the onset of myopia and perhaps also reduce its progression due to increasing dopamine level in the retina (i.e.^10^). Recently, interest was shifted towards the effects of spectral composition of light. Initial experiments in chickens had shown that blue light, moving the plane of focus more anteriorly and in front of the retina, also inhibits eye growth, while red light, associated with a longer focal length, made eyes grow longer^11,12^. Eye growth seemed to follow the focal plane position imposed by longitudinal chromatic aberrations (LCA). Results became the more complicated, the more animal models were studied. In particular, in tree shrews and monkeys, red light made the eyes shorter rather than longer^13,14^. Blue or near ultraviolet reduced myopia development that was induced by negative lenses in mice^15^, as well as myopia induced by frosted eye occluders in chickens^16^. Also, human eyes became transiently shorter in narrowband blue light and longer in narrowband green and red light^17^. These findings suggest that the retinal mechanisms for the control of eye growth need “reference points” across the entire visible spectrum. But how could the retina combine the information derived from the full visible spectrum? While it is possible that this could be done by comparing irradiances at different wavelengths and their temporal patterns^18^, it would be more intuitive that image focus at different wavelengths is compared. The idea is not new and has been discussed by others before^19,20^. Already Fincham in 1951 assumed that accommodation, the lenticular mechanism to focus the eye at different viewing distances, uses chromatic differences in focal planes^21^.

To find out whether the retina can indeed compare focus at different wavelengths, we filtered conventional movies in real time, using custom-developed software in Visual C++. Each pixel in the movies was convolved with the point spread function calculated from purely spherical defocus that originated from the human LCA function separately in the red, green, and blue image plane of RGB channels (see “Methods”)^22^. The calculated chromatic aberration function was either added to the natural chromatic aberration in the subject’s eyes or was presented inverted so that the total chromatic aberration function became flat at the cost of some blur in all channels (see “Methods”). In this case, the retina perceived similar blur at both ends of the spectrum.

The hypothesis was that red in better focus than blue would indicate to the retina that the eye is already too long, and that eye growth should be inhibited. Conversely, if blue is presented in better focus than red, it was expected that eye growth would increase. Of course, an important control experiment is that subjects also watched unfiltered movies with only their natural LCA. In the current experiment, as well as in many other studies, axial length is defined as the distance from the corneal apex to the vitreo-retinal interface. This distance can be measured with high repeatability using optical low coherence interferometry. If short-term changes occur, they trace back to changes in choroidal thickness. Therefore, the presented effects reflect changes in choroidal thickness. As in previous studies, the induced changes in the length of the eyes were tracked using low coherence interferometry, the Lenstar LS 900 (Haag Streit, Koenitz, Switzerland)^23,24^. It had been confirmed by others that the small changes in axial length trace back to changes in choroidal thickness (i.e.^25^) which, in turn, can predict future changes in eye growth and myopia development (i.e.^26^). Also in the current study, no changes were observed in corneal thickness (CT) or anterior chamber depth (ACD) in any of the experiments.

**Figure 1:**
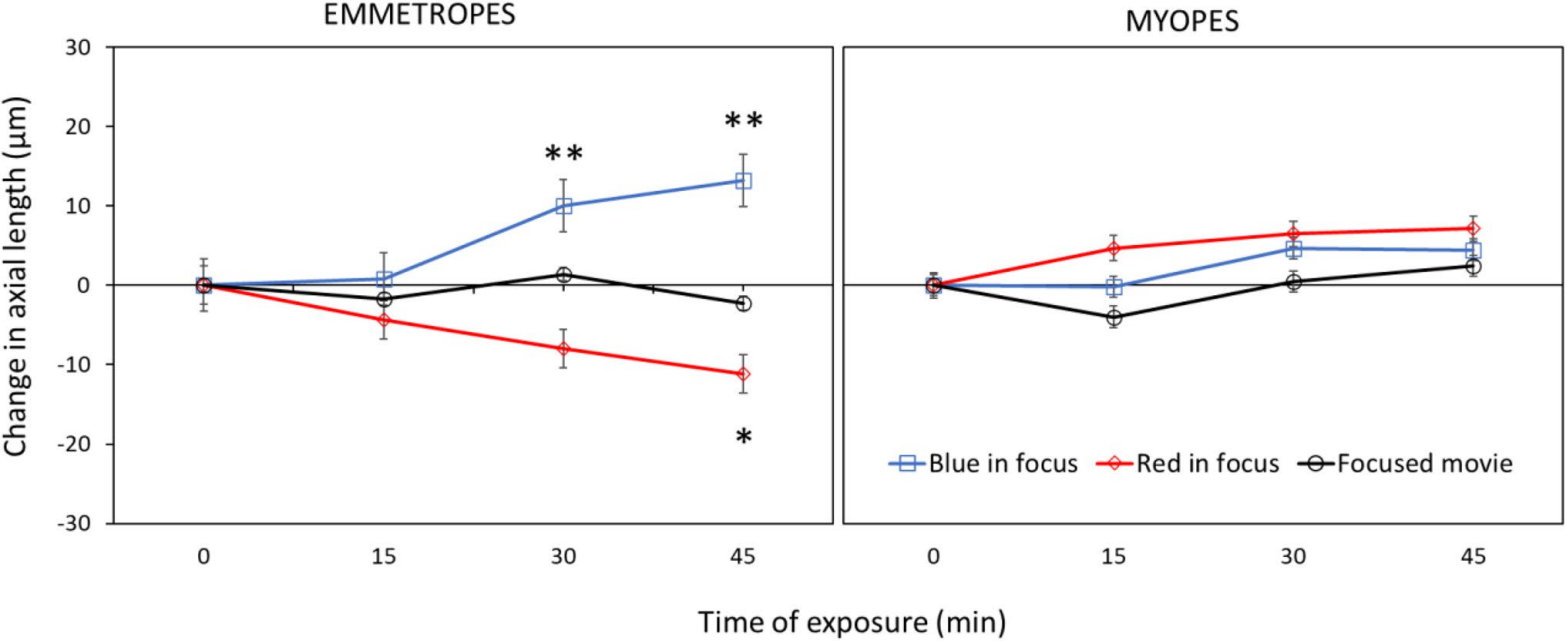
Changes in ocular axial length when subjects watched movies that were digitally filtered to present the red (“red in focus”) or blue (“blue in focus”) image plane in best focus. In emmetropic eyes, “red in focus” caused significant eye shortening (repeated measures ANOVA: p = 0.007) while “blue in focus” caused longer eyes (p = 0.0001). Strikingly, myopic eyes did not respond to these stimuli, indicating that they no longer detected the sign of defocus. Watching movies without chromatic filters did not induce changes in axial length, neither in emmetropes nor myopes (black lines). Data represents the averaged effect of all emmetropic (n = 20) and myopic (n = 15) participants. Error bars denote SEMs.

Results are shown in Fig. 1. Repeated-measures ANOVA revealed a significant influence of presented filters on change in axial length over time (F = 19.09, df = 2, p < 0.0001). With image focused more in red, occurred significant axial eye shortening over time (repeated-measures ANOVA F = 4.42, p = 0.007), as predicted by the hypotheses raised above (“red in focus” filter two-sided pairwise comparison: change in axial length after 45 min compared with a baseline: − 11.2 ± 14.1 μm, p = 0.013 (red lines); compared to control condition with unfiltered movies − 11.2 ± 14.1 μm vs. control − 2.2 ± 8.2 μm, two-sided pairwise comparison t = 2.42, df = 30, p = 0.01 (black lines)). Strikingly, no such changes were observed in myopic eyes (change after 45 min: + 7.1 ± 16.0 μm, two-sided pairwise comparison n.s., two-sided unpaired comparison to emmetropes: p = 0.001).

With calculated blur circles creating images more focus in blue and added to the natural LCA, the retinal image blur was imposed in both red and blue which induced significant eye elongation over time in emmetropes (repeated-measures ANOVA, F = 8.08, p < 0.001). Here, significant axial eye elongation was observed already after 30 min of stimulation (“blue in focus” filter two-sided pairwise comparison to the baseline: change in axial length after 30 min: + 10.0 ± 11.5 μm), and after 45 min (+ 13.2 ± 14.9 μm, both p < 0.01, blue lines). Axial eye length was also significantly increased compared to watching the unfiltered control movie (after 30 min: filter + 10.0 ± 11.5 μm vs. control + 1.4 ± 13.0 μm, two-sided pairwise comparison, t = 2.16, df = 35, p = 0.03; change after 45 min: filter + 13.2 ± 14.9 μm vs. control − 2.2 ± 8.2 μm, t = 4.01, df = 29, p = 0.0003). Moreover, there was a significant difference between change in axial length after watching “blue in focus” and “red in focus” movies after 45 min (+ 13.2 ± 14.9 μm vs. − 11.2 ± 14.1 μm, respectively, two-sided pairwise comparison, t = 5.29, df = 37, p < 0.0001).

Again, no changes were observed in myopic eyes (change in AL after 45 min: + 4.44 ± 18.1 μm, pairwise two-sided comparison, n.s.).

Neither emmetropes nor myopes showed any changes in eye length when they watched the unfiltered control movies (black lines; change in AL after 45 min: emmetropes − 2.2 ± 8.2 μm, myopes + 2.4 ± 13.0 μm, both two-sided pairwise comparison n.s.).

There was no influence of the time of day when the experiment was performed (morning or afternoon) or sex on changes in axial length in any part of the study (repeated-measures ANOVA with two within-subject factors: time of a day: p = 0.31, sex: p = 0.87, n.s.).

**Figure 2:**
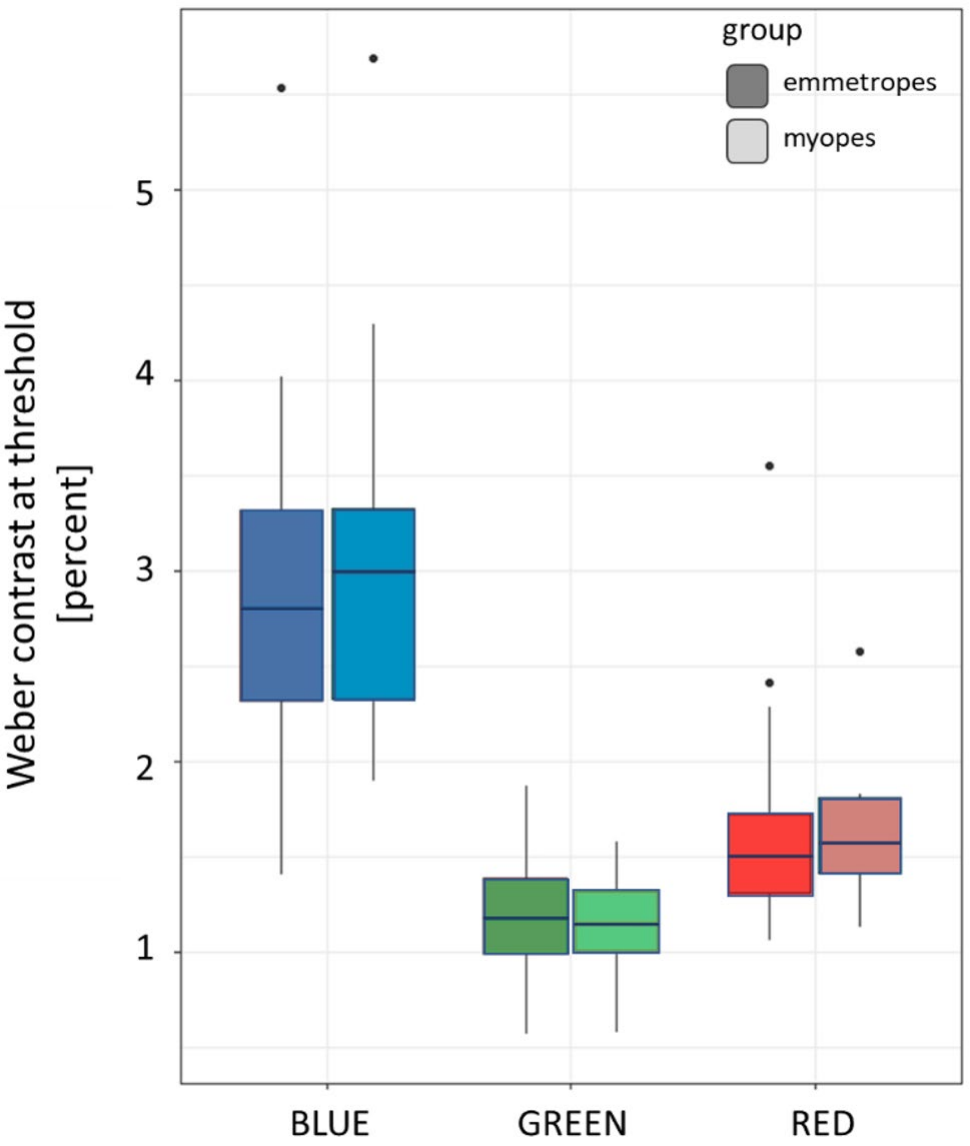
Weber contrast at the detection threshold in the blue, green, and red channel of the RGB format on the screen in emmetropic and myopic participants. No differences were observed between emmetropes and myopes.

An important question is now: why does the myopic retina no longer respond to chromatic defocus? A possible explanation could be that contrast sensitivity in the blue is reduced in myopic eyes to an extent that the retina can no longer determine the level of defocus. The change cannot be detected in the green or red because mid- and long wavelength sensitive photoreceptors mediate high acuity, and it is known that moderately myopic subjects have normal visual acuity and contrast sensitivity^27^. Software was developed in Visual C++ to measure contrast sensitivity in the blue, green and red RGB channel, using a 4-FAC procedure. Circular spatial patterns with a fundamental spatial frequency around 3–5 cyc/deg were presented on a gray background. Subjects had to select in which quadrant they saw parts of the circular pattern, using the arrow keys on the keyboard. Thresholds were automatically determined by the software using a staircase procedure (see “Methods”). All participants, including 20 emmetropes and 15 myopes, were tested (Fig. 2). Both emmetropic and myopic subjects needed higher contrast to detect the pattern in the blue (emmetropes vs. myopes: 2.92 ± 0.93 vs. 3.11 ± 1.07 percent Weber contrast, respectively, n.s.), compared to green (emmetropes vs. myopes: 1.16 ± 0.38 vs. 1.14 ± 0.25 percent Weber contrast, respectively, n.s.) and red (emmetropes vs. myopes: 1.66 ± 0.59 vs. 1.64 ± 0.37, respectively, n.s.). Therefore, we did not find loss in contrast sensitivity in the blue in myopic subjects that could explain why their eyes did not respond to chromatic defocus.

The question how the retina can detect the sign of defocus has a long history and a number of different theories were developed over time: (1) a role of peripheral astigmatism which is high in human eyes but low in chicken eyes^28^, (2) a role of high order aberrations which can generate asymmetrical changes in the image when defocus is imposed with different sign^6^, (3) a role of the Stiles-Crawford effect which may change the signal strength when oblique rays are reaching the photoreceptors^29^ (although a change in vergence resulting from 3 D of defocus changes the detected brightness by only less than a percent, and no sign of defocus information is generated), and finally (4) signals derived from longitudinal chromatic aberrations. The longitudinal chromatic aberrations appear most reliable because they contain information on the sign of defocus, are sufficiently large and require comparison of the output of only two photoreceptor types as found in most mammals (most are dichromats). The spatial resolution of blue cones in humans is about 5 cyc/deg, sufficient to detect a defocus of 1–2 D as needed for the mechanism^30^.

Because the blue image on the retina is already blurred by the natural LCA in the eye, we could not restore image sharpness in the blue by spatially filtering movies^31^. However, the red image could be blurred to a similar amount as the blue, so that the retina experienced similar blur in both spectral ranges (“blue in focus” filter), which we found to cause elongation of the eye. While blurry images are known to induce deprivation myopia, it is unlikely that this was the case here because images in the green were still reasonably well focused^32^. There must be a more specific mechanism behind—probably a comparison of sharpness in blue and red. We excluded in our study that loss of contrast sensitivity in the blue was responsible for the lacking response in myopes. An earlier study by Taylor et al. had found a minor decrease in contrast sensitivity in myopic subjects but the effects sizes were extremely small (p < 0.047 if one outlier of 44 observers was omitted)^33^. Since the differences between emmetropes and myopes in our study were prominent (Fig. 1), the findings by Taylor et al., are unlikely to explain the difference that we found between myopes and emmetropes. Therefore, other changes must have occurred in the retinal circuitry to compare the focus in the blue and red which need further studies.

After Rucker and Wallman^34^ had concluded from their experiments in chickens that “although previous work has shown that chromatic cues to defocus are not essential for lens-compensation, in that chicks can compensate in monochromatic light, our evidence implies that the eye may be able to infer whether the eye is myopic or hyperopic from the different chromatic contrasts that result from different signs of defocus.” Later, Gawne et al. analyzed whether S cone density in primates may be high enough to detect defocus in the blue that was imposed from longitudinal chromatic aberration^35^. They concluded that “the retinal spacing of the short-wavelength sensitive cones in many mammalian species is an evolutionarily ancient adaptation that allows the efficient use of chromatic cues in emmetropization.” A little later, Gawne et al. showed that this approach actually worked in tree shrews^36^. Tree shrews could be made more hyperopic simply by covering just one wall of their cages with an RGB screen that displayed a black and white pattern that was heavily low pass filtered in the blue channel. As a result, black-white edges had yellowish color fringes. The striking result of this experiment agreed with the hypothesis that, if blue is out of focus (and low pass filtered), it should tell the retina that the eye is already too long and further growth should be inhibited—and this is exactly what they found. Our current study is different in three regards (1) it is in humans (2) it involves movies instead of stationary patterns and (3) we simulated the chromatic blur exactly according to the human chromatic aberration function. It may be that the blur in the blue was less than in the study by Gawne et al.^36^, and in fact, the differently filtered movies did not appear very different to the subjects but still had the described effects on choroidal thickness.

Recently, it was found in tree shrews that low pass filtering of the blue image, induced more hyperopic refractions^36^. In this study, animals were kept in narrow cages which induced some degree of myopia based on their short viewing distances. Myopia could be prevented if one wall of the cage was covered with artificial visual targets that were low pass filtered only in the blue. Their findings relate to our findings in humans, although interactions with natural LCA were not analyzed. Gawne and Norton also developed a model how the tree shrews emmetropize based on a comparison of image focus in the S- and M-cone plane, which supports the hypothesis that LCA controls emmetropization^19^.

Our study relied on the assumption that participants were focused in the green, as described by Wyszecki and Stiles^37^, Benedi and Garcia et al.^31^, and Marcos et al.^22^. However, accommodation was not directly measured. But even if accommodation would have not focused perfectly on the mid-wavelength range, a number of recent studies have shown that defocus imposed by inaccurate accommodation has no effect on emmetropization (chicken:^38^; children:^39,40^).

Using LCA as a signal for emmetropization imposes interesting limitations. In human eyes, chromatic aberration spans over a dioptric range of maximally 2.5 D. If an eye becomes myopic, it may move out of the range where a comparison of sharpness in the blue and red provides a useful signal, because both may then be out of focus. While this could explain why myopia tends to progress when it is out of regulated range, it does not explain why optical correction does not restore the mechanism—and why refractions initially move out of the regulated range at all.

Nature rarely relies on one simple mechanism to control an important variable (in this case, refraction). Looking back at myopia research in the past, it becomes clear that several previous descriptions of “the mechanism of myopia” fell all short and described only one aspect of a large picture. Therefore, the current findings may also reflect only a part of emmetropization. Nevertheless, we still believe that our findings of functional deficiencies in the myopic retina move the question of myopia progression to another level. We are aware that there are several optical treatments that can reduce myopia progression, despite that we found that the myopic retina has reduced ability to detect positive defocus^23^. But is it really safe to assume that successful optical treatments (like DIMS designs or multifocal designs) work because they impose positive defocus on the retina? It could be any other kind of stimulation. Why should retinal image diffusing lens designs have any effect? They do not impose positive defocus. On the other hand, in our experiments we had a very few myopic subjects who responded to positive defocus or chromatic filtering just as emmetropes. A possible explanation is that their myopia was stable since long and that the emmetropization mechanism had partially recovered, keeping their refraction at an optimum with the optical correction in place.

## Conclusions

Our results demonstrate for the first time that the human retina uses the difference in focus in the blue and the red to determine the sign of defocus for emmetropization. Strikingly, this function is lost in myopes. While we had previously found that the myopic human retina has limited ability to respond to imposed positive defocus, the current results show now that the myopic retina has lost the ability to respond to longitudinal chromatic aberration.

## Methods

Thirty-five young adult participants (average age: 26 ± 3 years) were recruited. Prior to the experiments spherical equivalents of right eyes were measured without cycloplegia using a commercially available infrared photorefractor (PlusoptiX A12R binocular autorefractor, PlusOptix, Nuremberg, Germany). The experimental group included 20 emmetropes (7 males) with an average refractive error of − 0.38 ± 0.44 D and 15 myopes (5 males) with an average refractive error of − 3.41 ± 1.25 D. Subjects with astigmatism larger than 1D were excluded from the study. None of the subjects suffered from ocular pathologies other than moderate refractive errors. Informed consent was signed by each of the participants prior to the experiments. The study was approved by the Swiss Research Ethics Commission (EKNZ, reference 2020-01576) and met the requirements of the Declaration of Helsinki.

The participants were asked to watch binocularly a movie on a large TV screen (65 inches, LG OLED65C9, 4 K, 2019) at 2 m distance in a dark room. Myopic subjects wore their habitual corrections. The experiment was performed between 8 AM and 4 PM. The study protocol included 3 appointments on 3 separate days at the same time of the day for each individual participant. Day 1: watching the unfiltered movie served as a control, day 2: watching the movie digitally filtered to present the blue image sharp, but green and red low pass filtered according to the LCA function, and day 3: watching the movie digitally filter to present the red image sharp, and green and blue filtered according to the LCA function. Changes in axial length of the right eyes were measured during each session, before and after 15, 30 and 45 min of watching a movie. To avoid any additional factors that could influence the results, the same part of the movie was played for all conditions and for all participants. Moreover, the participants were asked to refrain from drinking coffee or smoking two hours prior to the experiment.

After the control session, each participant performed a contrast sensitivity test for red, green, and blue spatial stimuli, displayed on gray background.

### Ocular biometry

Axial length (measured as the distance between the outer surface of the cornea and RPE), corneal thickness (CT) and anterior chamber depth (ACD) of the right eyes were assessed by using a low coherence interferometer with auto positioning system (Lenstar LS 900 Haag-Streit, Koeniz, Switzerland). Measurements were done before and immediately after every 15 min of watching chromatically filtered or the control movies. The average data from three repeated measurements were used. Due to the auto positioning system of the device and an extensively trained operator, standard deviations were typically around 5 µm for the axial length measurements.

### Chromatic spatial filtering

**Figure 3:**
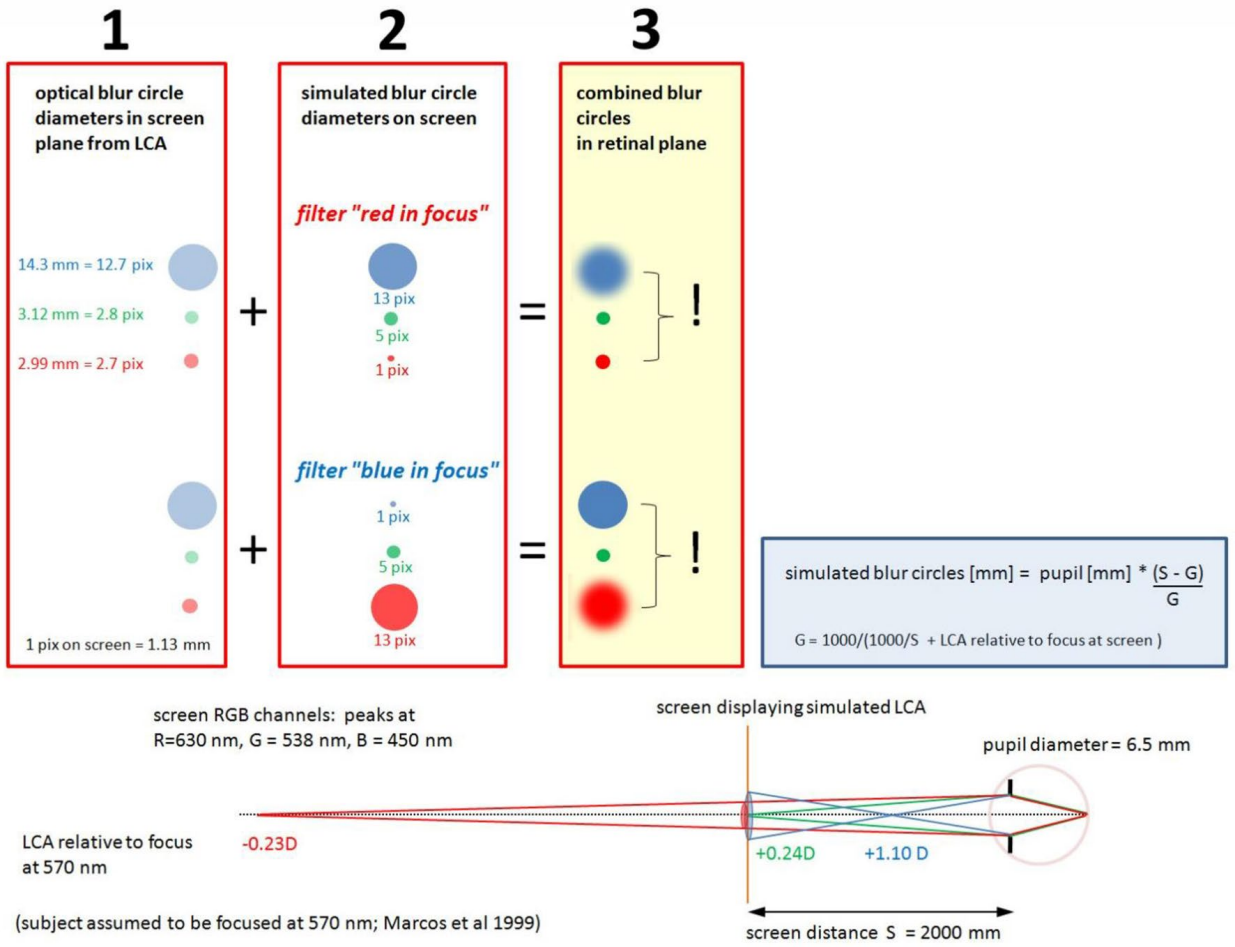
Subjects watched movies on the screen at 2000 mm distance. As proposed by Marcos et al.^22^, subjects are assumed to be in best focus at 570 nm (see illustration at the bottom of the Figure). According to the LCA function in human subjects (shown below in Fig. 4), the red channel in RGB (630 nm) is defocused hyperopically by − 0.23D, the blue channel (450 nm) myopically defocused by + 1.10D while the RGB green channel (538 nm) is slightly myopically defocused by + 0.24D. The respective calculated blur circles are shown in red box 1. Movies were filtered as shown in red box 2. With the filter “red in focus”, the image of the red channel remained untouched while green and blue were spatially filtered according to the LCA. With the filter “blue in focus”, the image of the blue channel remained untouched while red and green were spatially filtered according to the LCA. Combining box 1 and 2, the blur circles on the retina could be changed so that either blue was low pass filtered and red was sharp, or both were low pass filtered by combining natural LCA with the calculated blur circles accordingly to the LCA function (box 3).

Movies were chromatically filtered in real-time by custom-developed software written in Visual C++, which allowed to individually blur each of the RGB channels (red, green, or blue). With a video format of 1280 × 720 pixels, filtering was possible at about 25 Hz (Hz) frame rate. To apply the same frame rates to all experimental conditions, also the unfiltered control movie was run through the software. Spatial filtering had no effect on the average pixel values in the three RGB channels which were continuously shown by the software. Therefore, the appearance of color of the movies did not change during filtering. Two chromatic filters were used: “red in focus” where red pixels were untouched, while green and blue pixels were blurred (Fig. 3), and “blue in focus” where blue pixels were untouched while green and red pixels were blurred (Fig. 3). The combination of blur circle generated by natural LCA (Fig. 3, box 1) and calculated LCA (Fig. 3, box 2) let to blur circles on the retina that either caused low pass filtering in the blue and a sharp image in the red (filter “red in focus”) or low pass filtering in the red, but combined with LCA blur in the blue (filter “blue in focus”) (Fig. 3, box 3). The important difference was that once red appeared sharp and blue blurry (“red in focus”), and once both appeared blurry (“blue in focus”). If the retina performs a comparison of sharpness in blue and red, the induced changes in axial eye length should be different in both cases. For the calculations of the blur circles, a pupil size of 6.5 mm was used which was the average pupil size of all subjects. There was no difference between emmetropic and myopic subjects in our sample. The calculations assumed that subjects were in best focus at 570 nm as proposed by Marcos et al.^22^. The calculated blur circles diameters can be readily converted into angular units. In the human retina, 1 deg in the visual field maps to 290 µm on the retina. Accordingly, a blur circle diameter of 14.3 mm at 2000 mm distance corresponds to 24.6 arcmin, using that 1 pixel on our large TV screen had a diameter of 1.13 mm, equivalent to 1.94 arcmin.

**Figure 4:**
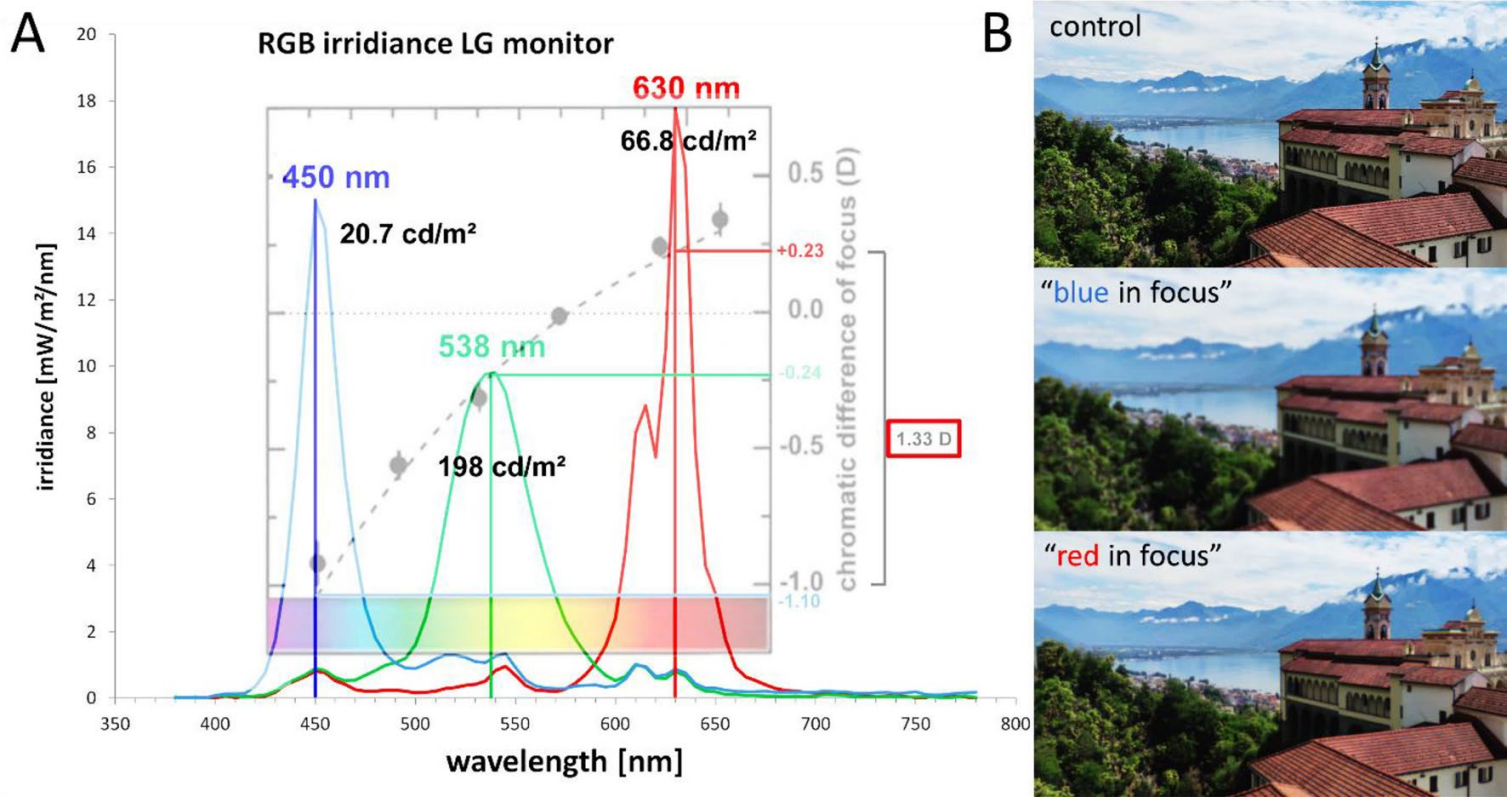
(**A**) Spectral irradiances of the blue, green and red channel on the RGB screen, and the respective peak wavelengths, as measured with a commercial photospectrometer (Gossen, MAVOSPEC BASE, Nuremberg, Germany). The dotted line shows the chromatic defocus function, relative to 570 nm, as provided by Marcos et al.^22^. Black numbers indicate the luminance, separately measured with a Minolta candela meter (LS-100, Minolta Camera Co., LTD, Tokyo, Japan). Point spread functions for the chromatic blur were calculated relative to 570 nm (Fig. 3). (**B**) Illustration of images filtered with the “blue in focus” and “red in focus” filter. Note that image with “blue in focus” filter appears more blurry than the image with “red in focus” filter because the human visual system uses M and L cones for high acuity tasks and blur in red and green has higher impact of visual acuity.

The human chromatic aberration function and the derived chromatic defocus used to calculate the blur circle diameters in Fig. 3 is shown in Fig. 4 (after Marcos et al.^22^).

**Figure 5:**
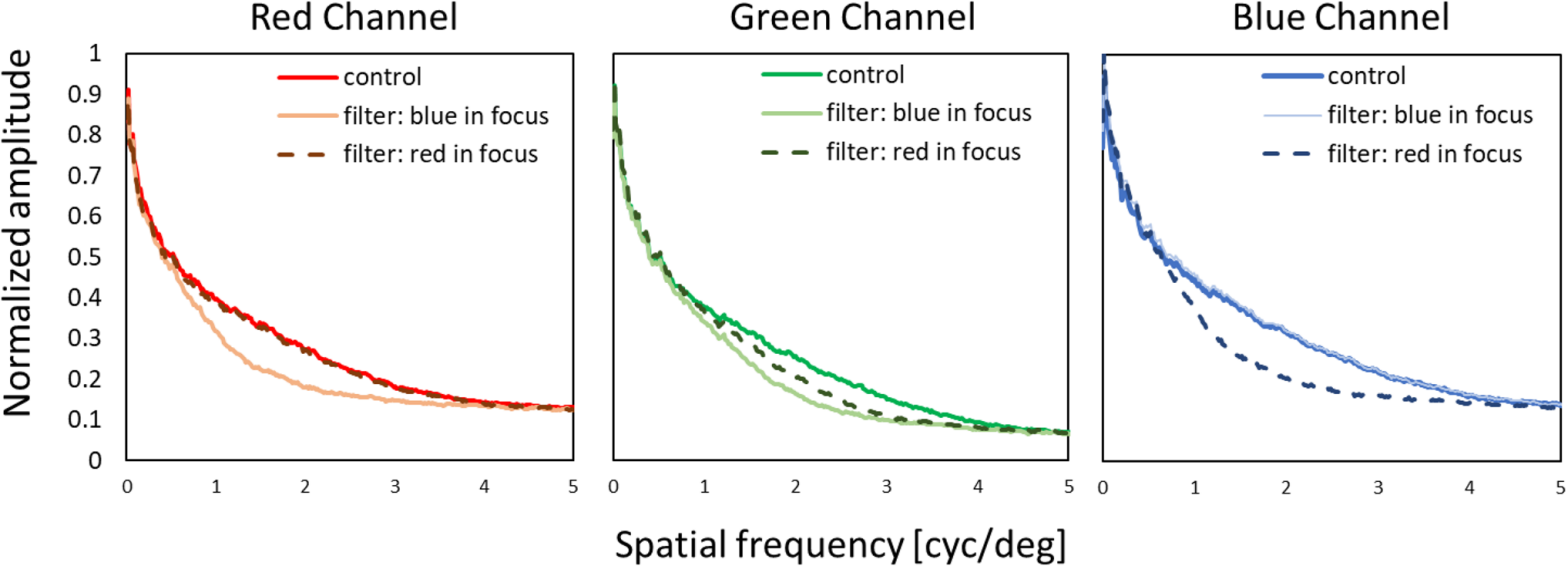
Spatial frequency spectra determined by Fourier analysis of the control, “blue in focus” and “red in focus” filtering condition.

The modulation transfer functions in red, green, and blue were also determined. We used a RGB camera (DFK 33UX290, Imaging Source, Germany, 1920 × 1080 px resolution) with similar optical parameters as the human eye with 6.5 mm pupil (16 mm focal length, f/# 2.6) and 16 mm lens (M1614-MP2, Computar, Japan) to obtain spatial frequency spectra for control, “blue in focus” and “red in focus” movies. Pictures of the three conditions were taken from the screen showing the movies at 2 m distance. RGB channels were separated, and Fourier analysis performed with publicly available software (ImageJ, National Institutes of Health [NIH], http://imagej.nih.gov/ij/). Subsequently, one-dimensional plots were created by rotational averaging of the two-dimensional spatial frequency spectra, using custom-developed software written in Visual C++. Averaged pixel values were normalized to the maximum of 1 and the minimum of 0 for each RGB channel in every experimental condition, as shown in Fig. 5.

### Measurement of contrast sensitivity in RGB red, green, and blue

**Figure 6:**
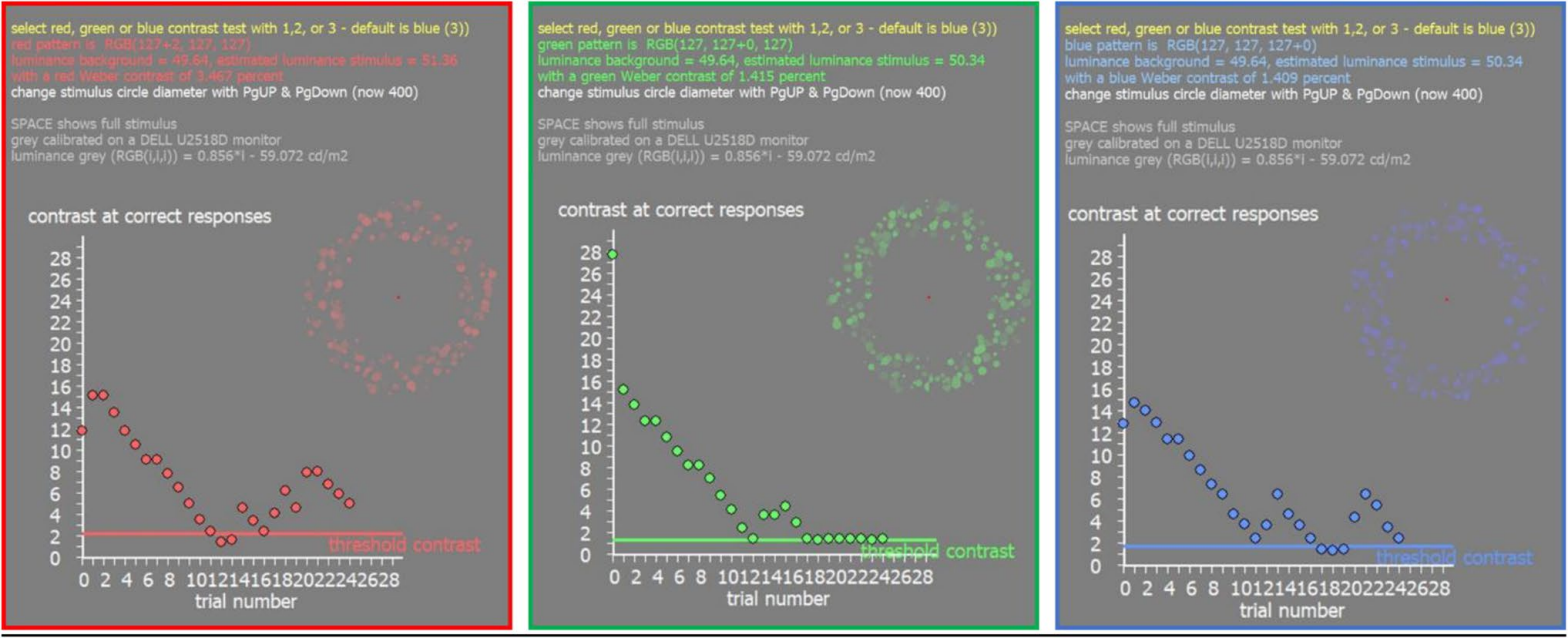
Illustration of the screen output of the software to measure contrast sensitivity in R, G, and B. In the top right of each picture, the appearance of the stimuli is illustrated but during the real test, they cover a major part of the screen (diameter of the circular pattern 400 pixel). In the bottom, the output of the staircase procedure is shown.

Custom-developed software written in Visual C++ was used to display annular hemi-circular patterns of dots of varying size on a grey screen (RGB (127,127,127) either in red, green, or blue (DELL U2518D, 25″, 1920 × 1080 px resolution) at 50 cm distance. Chromatic hue was generated by simply increasing the individual RGB values of the dots in steps of one (i.e., red RGB (128,127,127). The pattern appeared randomly on the top, right, down, or left side. Participants had to indicate where they see the pattern using the arrow keys (up, down, right, or left) of the keyboard. If the answer was correct, the pixel brightness value in the tested channel was reduced by 1, moving its color closer to grey. When subjects made wrong choice, pattern contrast was increased by 3-pixel values and then reduced again when the selection was correct. After 25 selections, the lowest contrast of 5 correct choices were averaged and used as a measure of the detection threshold. Contrast of the pattern was measured as Weber fraction after an initial individual calibration of the screen luminance in R, G, and B with a Minolta candela meter. Background luminance of the screen was 49.64 cd/m^2^ and starting Weber contrast was about 10% for all color patterns. Results from three repeated tests were averaged and served as a threshold value of Weber contrast for each individual participant, separately for each color (Fig. 6).

### Statistical analyses

All statistical calculations were performed using the publicly available software environment R (version R 4.1.0; R Core Team, R Foundation for Statistical Computing, Vienna, Austria). Data from each refractive group are presented as the averages from either all myopic or emmetropic subjects, together with the standard errors. The QQ plots were used to confirm that the axial length data were normally distributed. Changes in axial lengths during watching digitally filtered or control movies were analyzed using a repeated measures analysis of variance (ANOVA) with two within-subjects factors of time and type of filter (“blue in focus”, “red in focus”, control), followed by a post hoc test with Bonferroni correction. In addition, influence of two between-subjects factors (time of day when experiment was performed: morning or afternoon, and sex) was calculated for each tested condition. Changes in CT + ACD before and after 45 min of stimulation were analyzed using a paired Student’s T-test. The results of the contrast sensitivity measurements were analyzed separately for blue, green, and red by using Wilcoxon test to compare differences between myopic and emmetropic eyes.

## Data Availability

The datasets used and/or analysed during the current study available from the corresponding author on reasonable request (Dr. Barbara Swiatczak barbara.swiatczak@iob.ch). The software to simulate LCA in real time in images provided by a (laptop) camera can be downloaded here (exe file and libraries only) https://www.dropbox.com/sh/u1ppxub1ef3exnp/AAAH2NwA03vc7T2NpoWf9l4ma?dl=0 (after starting the “RGB LCA with text.exe” first select your camera and select MJPEG(1280x720) video format). The software to measure contrast sensitivity in R, G and B can be downloaded here (exe file only): https://www.dropbox.com/s/qy2xejy5p9zb256/blue%20cone%20contrast.exe?dl=0.
